# Changes in Maternal Heart Rate Variability and Photoplethysmography Morphology after Corticosteroid Administration: A Prospective, Observational Study

**DOI:** 10.3390/jcm13082442

**Published:** 2024-04-22

**Authors:** Maretha Bester, Thomas J. Nichting, Rohan Joshi, Lamyae Aissati, Guid S. Oei, Massimo Mischi, Judith O. E. H. van Laar, Rik Vullings

**Affiliations:** 1 Department of Electrical Engineering, Eindhoven University of Technology, 5612 AZ Eindhoven, The Netherlands; 2 Patient Care and Monitoring, Philips Research, 5656 AE Eindhoven, The Netherlands; 3 Department of Obstetrics and Gynecology, Máxima Medical Centrum, 5504 DB Veldhoven, The Netherlands; 4 Faculty of Health, Medicine and Life Science, Maastricht University, 6229 ER Maastricht, The Netherlands

**Keywords:** corticosteroids, betamethasone, pregnancy, maternal health, heart rate, heart rate variability

## Abstract

**Background**: Owing to the association between dysfunctional maternal autonomic regulation and pregnancy complications, assessing non-invasive features reflecting autonomic activity—e.g., heart rate variability (HRV) and the morphology of the photoplethysmography (PPG) pulse wave—may aid in tracking maternal health. However, women with early pregnancy complications typically receive medication, such as corticosteroids, and the effect of corticosteroids on maternal HRV and PPG pulse wave morphology is not well-researched. **Methods**: We performed a prospective, observational study assessing the effect of betamethasone (a commonly used corticosteroid) on non-invasively assessed features of autonomic regulation. Sixty-one women with an indication for betamethasone were enrolled and wore a wrist-worn PPG device for at least four days, from which five-minute measurements were selected for analysis. A baseline measurement was selected either before betamethasone administration or sufficiently thereafter (i.e., three days after the last injection). Furthermore, measurements were selected 24, 48, and 72 h after betamethasone administration. HRV features in the time domain and frequency domain and describing heart rate (HR) complexity were calculated, along with PPG morphology features. These features were compared between the different days. **Results**: Maternal HR was significantly higher and HRV features linked to parasympathetic activity were significantly lower 24 h after betamethasone administration. Features linked to sympathetic activity remained stable. Furthermore, based on the PPG morphology features, betamethasone appears to have a vasoconstrictive effect. **Conclusions**: Our results suggest that administering betamethasone affects maternal autonomic regulation and cardiovasculature. Researchers assessing maternal HRV in complicated pregnancies should schedule measurements before or sufficiently after corticosteroid administration.

## 1. Introduction

The detection of pregnancy complications before the onset of their detrimental symptoms is a persistent challenge in perinatology. The early detection of complications allows for pharmaceutical or lifestyle interventions, as well as improved monitoring, which leads to improved perinatal outcomes [1,2].

A promising monitoring tool for detecting the early onset of deterioration in maternal health is the assessment of maternal heart rate variability (mHRV) [3,4,5]. Given that changes in heart rate (HR) are regulated by the autonomic nervous system (ANS) [6]—and further considering that women with pregnancy complications have altered ANS activity compared to their healthy counterparts [7,8,9]—abnormalities in mHRV may be predictive of pregnancy complications. The use of mHRV to screen for abnormalities requires a clear understanding of how mHRV is altered during complicated pregnancies. Consequently, several researchers have investigated how mHRV is altered in women with pregnancy complications [3,9,10].

However, investigations into the mHRV of women with complicated pregnancies have been hindered by the routine administration of obstetric medications, such as tocolytics, beta-blockers, and corticosteroids, which are administered to women upon the diagnosis of complications. As the effect of these medications on mHRV is largely unknown, it is uncertain to which degree changes in HRV in this population reflect autonomic dysregulation associated with pregnancy complications, as opposed to merely reflecting the confounding effect of these medications [10,11,12,13].

While the impact of all routine obstetric medications warrants investigation, the drugs most administered to pregnant women with complications are corticosteroids. Corticosteroids—specifically, betamethasone—are maternally administered for fetal lung maturation in anticipation of preterm labor, which is a typical concern in cases of early pregnancy complications [14]. Researchers have already shown that administering betamethasone invokes changes in fetal HR and HRV [15,16,17]. Furthermore, betamethasone is a glucocorticoid, with the latter being a class of drugs known to activate the cardiac stress response [18]. Therefore, we hypothesize that mHRV also changes in response to betamethasone administration.

Consequently, we set out to understand whether antenatally administered corticosteroids affect the maternal physiology. To this end, we aim to quantify the effect of antenatally administered corticosteroids (specifically, betamethasone, which is commonly used in antenatal care) on mHRV based on continuous photoplethysmography (PPG) recordings performed in women hospitalized with pregnancy complications. Furthermore, as a sub-analysis, we also examine the changes in the morphology of the PPG pulse wave signal after betamethasone administration to gain further insight into the effect of corticosteroids on maternal physiology.

## 2. Materials and Methods

The Materials and Methods section details the prospective, observational study performed to collect PPG data from women who were administered betamethasone (Section 2.1). Thereafter, the analyses performed are detailed, specifically, a main analysis and a sub-analysis to determine the effect of corticosteroids on mHRV (Section 2.2) and the morphology of the maternal PPG pulse wave (Section 2.3), respectively. Finally, the statistical analysis of the data is described (Section 2.4). All processing was carried out in Python (PSF, Wilmington, DE, USA).

### 2.1. Study Design

Pregnant women admitted to the obstetric high-care unit at Máxima Medical Center (Máxima MC) who had an indication for betamethasone (Celestone Chronodose^®^, Schering AG, Berlin, Germany; 2 doses of 12 mg intramuscularly, 24 h apart) but had not yet received their second injection were invited to participate in this study. Such admissions are typically performed with urgency as these patients are at risk of preterm birth. Consequently, this study—of which the protocol has been published [19]—was designed to have a low impact on clinical workflow. To this end, enrolled women received a wristband-like device to wear for the duration of their hospitalization which recorded PPG and tri-axial accelerometer data. This device (Philips Data Logger, Philips Research, Eindhoven, The Netherlands) continuously recorded the data at 32 Hz and offloaded it via Bluetooth to an accompanying mobile phone kept in the patient’s room, which served as a data storage device. All women were at least 18 years old and without medical equipment or tattoos on their wrists that could obstruct data collection with the PPG device. Inclusion and exclusion criteria are further outlined in Table 1.

Patient metadata (e.g., complication, age, and medications along with the timing of their administration) were collected from patient medical records. The co-administration of medications was unavoidable since this was part of the standard treatment protocol [20,21,22]. To attenuate contractions in cases of threatened preterm labor, tocolytics such as nifedipine or atosiban were administered. Antibiotics such as azithromycin or penicillin were administered to prevent infections, for example, in the case of preterm rupture of membranes. Additionally, women with hypertensive disorders of pregnancy could receive antihypertensive medications; specifically, methyldopa or labetalol. Magnesium sulfate can also be administered in cases of preeclampsia or for fetal neuroprotection.

All participants gave informed oral consent followed by delayed written consent, as approved by the Medical Ethics Committee of Máxima MC, Veldhoven, The Netherlands. The approval for the study is waived by the Medical Ethics Committee of Máxima MC, Veldhoven, The Netherlands (N19.112; 2 December 2019) and the study is registered in the Dutch Trial Register (NL8204; 6 December 2019). All methods were carried out in accordance with the Declaration of Helsinki, with the relevant guidelines and regulations.

### 2.2. Main Analysis: The Effect of Administering Betamethasone on mHRV

**Figure 1:**
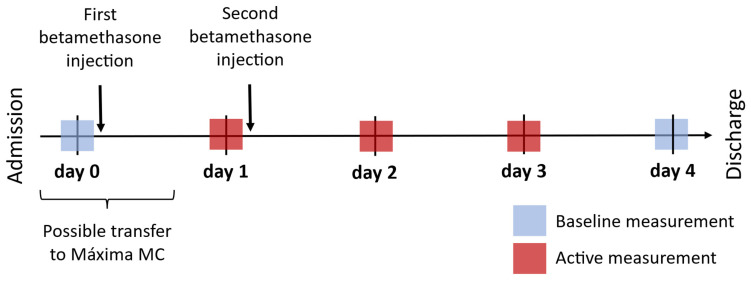
Five-minute measurements are selected preceding (if possible) and following the administration of betamethasone to assess the effect of this medication on mHRV and PPG pulse wave morphology. This figure illustrates the timing of the measurements on *days* 0 to 4. The measurement from *day* 0 is the baseline measurement; if not available, the measurement from day 4 can be used as a baseline measurement. Note that measurements on *day* 0 and *day* 1 have to precede the first and second injections of betamethasone, respectively.

The main purpose of this work is to determine the effect of betamethasone on mHRV using a within-subject (paired) analysis. We compare HRV features at baseline (i.e., without the influence of betamethasone) against HRV in the days following betamethasone administration, as outlined in Figure 1. Since women who receive corticosteroids in the antenatal period are heterogeneous in terms of their pregnancy complications, age, gestational age, number of fetuses, etc., a within-subject analysis was chosen to minimize the effect of these differing characteristics [9,23]. Moreover, this choice was also made to reduce the impact of circadian rhythms on the analysis [24]; since betamethasone can be administered at any time, one participant may receive her first betamethasone injection at noon while another receives hers at midnight.

In the following sections, we outline the timing and selection of the PPG segments used for this analysis, the preprocessing of these segments for determining HRV, and the calculation of the HR and HRV features, with the latter comprising time-domain, frequency-domain, and non-linear features.

#### 2.2.1. Timing of Measurements

For calculating HRV, five-minute PPG segments were identified from the continuous PPG recordings for each *day*, as outlined in Section 2.2.2. Acquiring a baseline measurement before the participant receives betamethasone is challenging, partly because corticosteroids are typically administered with urgency. Moreover, given that Máxima MC (the study site) is a tertiary teaching hospital, women are often transferred here after initial diagnosis and treatment at their primary hospital. Consequently, most women would have already received their first betamethasone injection before arriving at Máxima MC. To this end, for the main analysis concerning mHRV, we defined the baseline measurement as either before the first betamethasone injection (noted as *day* 0 in Figure 1) or sufficiently thereafter. In the latter case, *day* 4 was used as the baseline measurement, based on the pharmacokinetics of betamethasone (a biological half-life of 36–59 h [25,26]) and the duration of the drug’s effect on fetal HR and HRV (approximately 48 h [15,16,17]). This decision is further detailed in the study protocol [19]. Additionally, active measurements are acquired approximately 24 h, 48 h, and 72 h after the first injection (±4 h), i.e., *days* 1, 2, and 3. Note that measurements on *day* 0 and *day* 1 had to precede the first and second injections of betamethasone, respectively.

#### 2.2.2. Segment Selection 

Furthermore, even though participants in this study were hospitalized for the duration of the study, the study setup resembled free-living conditions as the women were free to move their extremities as well as remove and reattach the PPG wristband. Consequently, motion artifacts and periods of sensor detachment were common in the data. Considering these limitations, as well as the constraints regarding the timing of the measurements, the data segments to be included were manually selected through exploratory data analysis. A single, contiguous five-minute segment was chosen for each *day*. Segments used for the analysis had to have no more than 20% of the inter-pulse intervals (IPIs) discarded during preprocessing, as defined in Section 2.2.3.

In cases where a measurement for *day* 0 was available, the measurement moment was chosen to be as close to one hour before the first betamethasone injection as possible, considering the timing constraints for the subsequent measurements (i.e., within 24 h, ±4 h) as well as the requirement for data quality discussed in the preceding paragraph. If *day* 0 was not available, *day* 1 was similarly chosen with regard to the second betamethasone injection, again with the consideration of the timing of subsequent measurements and data quality.

#### 2.2.3. Preprocessing of PPG Measurements for HRV Analysis

For the HRV analysis, the IPI values are calculated as the difference between two contiguous waveform troughs, which are detected using a published algorithm [27]. IPIs were disregarded when considered physiologically implausible, i.e., when shorter or longer than 0.4 or 2.0 s, respectively, or when differing from their preceding interval by more than 20%. For the calculation of HRV features that relate to the frequency domain or complexity, a continual time series is needed, and subsequently, missing IPIs are replaced with cubic spline interpolation when calculating these features.

#### 2.2.4. Determining HR and HRV Features

First, the average of all the IPIs from each segment was determined and then converted to beats per minute (bpm) to obtain the HR. Thereafter, several HRV features were calculated. In the time domain, the standard deviation of the IPIs (SDNN) is calculated to capture overall HRV. Additionally, two features that capture parasympathetic activity were calculated, namely, the root mean square of the successive differences of the IPIs (RMSSD) and the percentage of consecutive IPIs differing by more than 50 ms (pNN50) [6,28].

Furthermore, HRV can also be assessed in the frequency domain. To this end, we calculate the following features: the total power (TP) in the frequency domain, the power in the high-frequency band of 0.15–0.40 Hz (HF), the power in the low-frequency band of 0.04–0.15 Hz, and the ratio between the two (LF/HF). While LF is influenced by both branches of the ANS (i.e., both parasympathetic and sympathetic activity), HF captures mainly parasympathetic activity. Consequently, LF/HF may provide information on the balance between the two branches by capturing what is referred to as the sympathovagal balance [6,28]. However, experts urge that this parameter should be interpreted cautiously [6].

We further determine some non-linear features of HRV. We use a Poincaré plot, which is a scatter plot of each IPI plotted against its predecessor. An ellipse is then fitted to this plot and two standard descriptors, namely SD1 and SD2, are calculated to represent the short- and long-term HRV, respectively. Similar to LF/HF, the ratio of these—SD1/SD2—offers a window into sympathovagal balance [6,29], as short-term and long-term variability are primarily modulated by parasympathetic and sympathetic activity, respectively. Additionally, we assess the complexity of the signal represented by the IPIs with sample entropy (SampEn) [30,31]. SampEn quantifies the conditional probability that two epochs, which are similar within a tolerance *r* for a window length *m*, will remain similar when including the next data point (i.e., the next IPI). The parameters *m* and *r* were set to 2 and 0.2 times the standard deviation of the IPIs. Lower SampEn indicates a more predictable time series, i.e., a time series with higher regularity. Finally, we use detrended fluctuation analysis (DFA) [32], which is used to quantify the self-similarity of IPIs over time. A healthy HR pattern is not completely random. However, a healthy HR is also not fully predictable, rather, the HR time series contains trends that will repeat over different timescales. Using *α*_1_, the short-term fractal scaling exponent of the DFA, which represents the correlation in the IPIs over 4–16 heartbeats, we can capture this characteristic of the HR. An *α*_1_ of 1 suggests a high level of self-similarity. As *α*_1_ decreases, the HR time series becomes more predictable [30,33].

### 2.3. Sub-Analysis: The Effect of Administering Betamethasone on the Morphology of the PPG Pulse Wave

The effect of betamethasone on the morphology of the PPG pulse wave (hereafter referred to as morphological features) has not previously been investigated to our knowledge. Consequently, for the sub-analysis—i.e., investigating the impact of betamethasone on maternal morphological features—no prior information is available to support the use of day 4 as a suitable baseline measurement (as seen in Figure 1). Subsequently, we do not group day 0 and day 4 as baseline measurements but rather perform an unpaired analysis across all five days to examine the impact of betamethasone on the pulse wave. The same segments used in the HRV analysis (see Section 2.2.1 and Section 2.2.2) are also used for this analysis.

#### 2.3.1. Preprocessing of PPG Measurements for Analysis of Morphological Features

**Figure 2:**
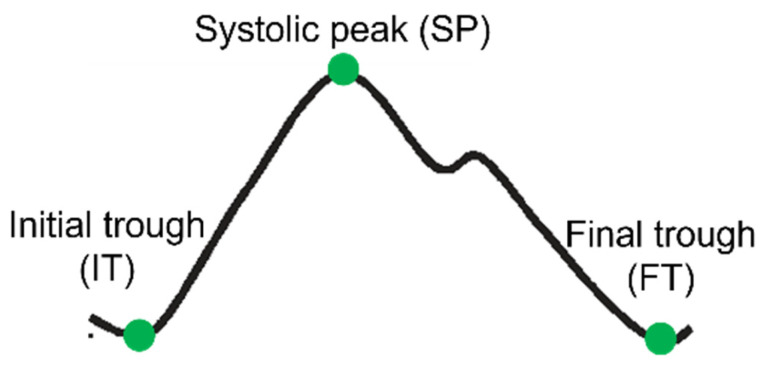
PPG pulse wave with relevant fiducial points.

To determine the PPG features assessed in this study, the PPG pulse wave needs to be segmented to identify the relevant fiducial points, namely the initial trough (IT), systolic peak (SP), and final trough (FT), as seen in Figure 2. To this end, NeuroKit2—a publicly available Python package for analyzing physiological signals [34]—was used. Pulses for which these fiducial points were not detected were excluded from the analysis, as these points are needed to calculate the features described in Section 2.3.2.

#### 2.3.2. PPG Morphology Features

**Figure 3:**
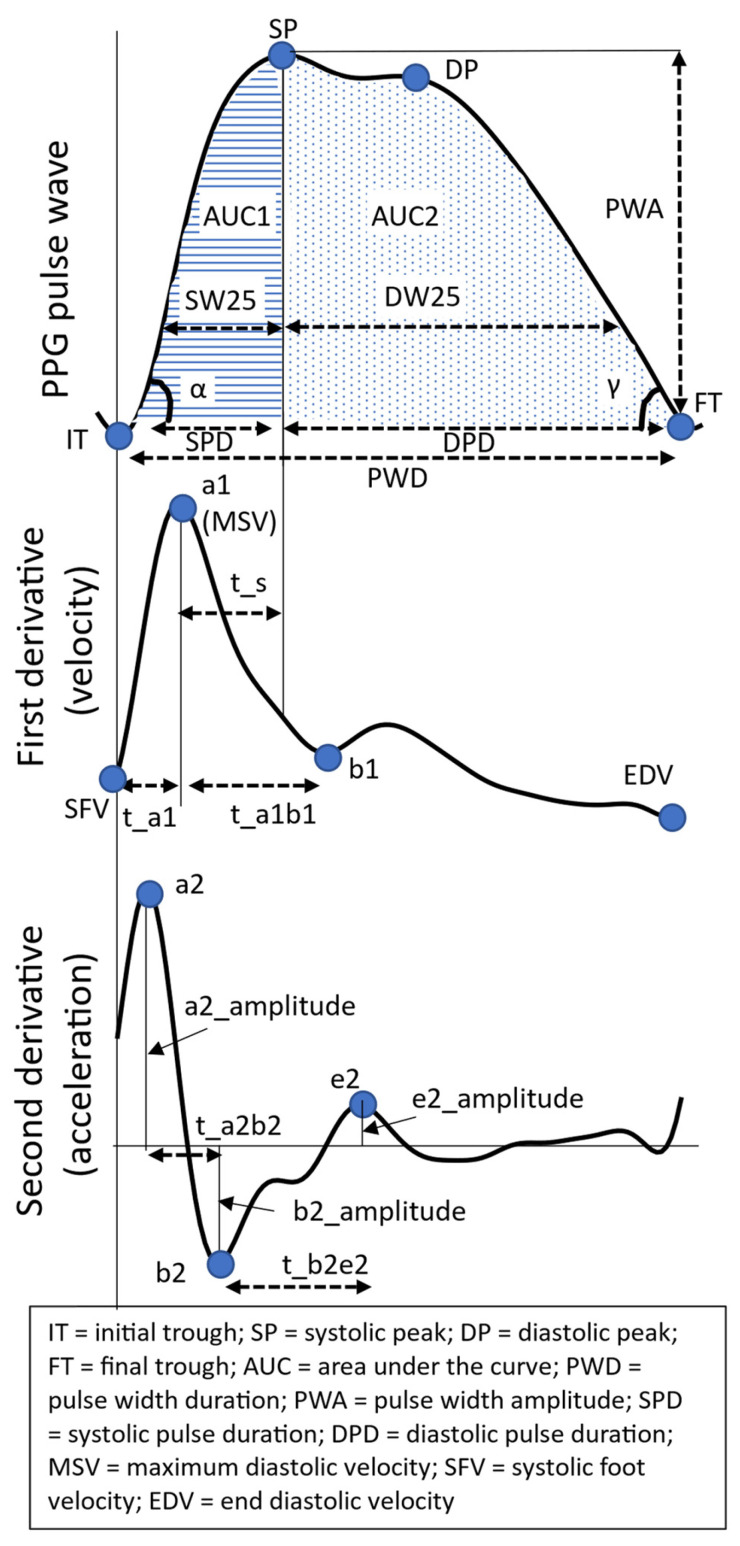
The top figure represents the PPG waveform, followed by the first and second derivatives of the waveform. Aspects of these waveforms relevant to the PPG morphology features listed in Table 2 are indicated in the figures.

The PPG pulse wave is a reflection of the blood flow through the vascular bed [35]. The initial rising edge of the pulse wave (i.e., from IT to SP, Figure 2) reflects the systolic phase of the heartbeat, while the falling edge corresponds to the diastolic phase (SP to FT). By assessing features describing the morphology of this pulse wave, we gain insight into vascular tone and by proxy its regulation via the ANS. While the exact mechanisms originating the different components of the PPG pulse wave are not known, these features are generally considered to provide valuable physiological information [35]. The features that are used to describe the waveform are shown in Figure 3 with corresponding descriptions in Table 2 [36,37,38]. Note that in addition to describing the geometry of the PPG pulse wave, features detailing the first and second derivatives of the PPG pulse wave are also often used.

### 2.4. Statistical Analysis

A sample size of 61 was calculated using a two-sided *t*-test for a confidence interval of 95% and a power assumption of 80% (see protocol for further details [19]). As physiological data are typically non-parametrically distributed, we perform a non-parametric analysis throughout. As discussed in Section 2.2, a within-subject analysis is performed to compare HRV across the different *days* using Friedman’s test with Dunn’s post hoc test. The former provides information as to whether statistically significant changes occur across the four *days* analyzed while the latter reveals whether statistically significant differences exist between specific *days*, e.g., *day* 1 and *day* 3. Bonferroni correction was implemented to control for family-wise error. Furthermore, an unpaired analysis was performed to investigate changes in morphological features using a Kruskal–Wallis test with a Dunn’s post hoc test and Bonferroni correction to control for family-wise error. Note that the statistical analysis that was presented in the protocol has been updated in this work; the authors believe the analysis presented here is the more appropriate one. A value of *p* < 0.05 was considered statistically significant. Furthermore, effect sizes are reported along with statistical significance using Cohen’s *d*, where 0.2 amounts to a small effect, 0.5 to a medium effect, and 0.8 to a large effect. We further perform a bootstrapping procedure (10,000 iterations) and report the subsequent mean *d*-value along with the 95% confidence intervals (CIs), as is appropriate in non-parametric analyses [39].

## 3. Results

First, we describe the included participants in the study (Section 3.1). Thereafter, we present the results from the within-subject analysis performed to determine the effect of administering betamethasone on mHRV (Section 3.2). Following this, the results from the sub-analysis which investigates the effect of this drug on morphological features are given (Section 3.3). Results are presented as plots of the median values and interquartile ranges of the features; appropriate statistics are displayed in the figures.

### 3.1. Study Group

A total of 143 women were enrolled in the study between July 2020 and January 2022. Of these, 61 women had sufficient measurements to be included in the analysis. Participant demographics are outlined in Table 3. Eight women had a *day* 0 measurement to use for the baseline measurement, while *day* 4 was used for the others.

### 3.2. Main Analysis: The Effect of Administering Betamethasone on mHRV

**Figure 4:**
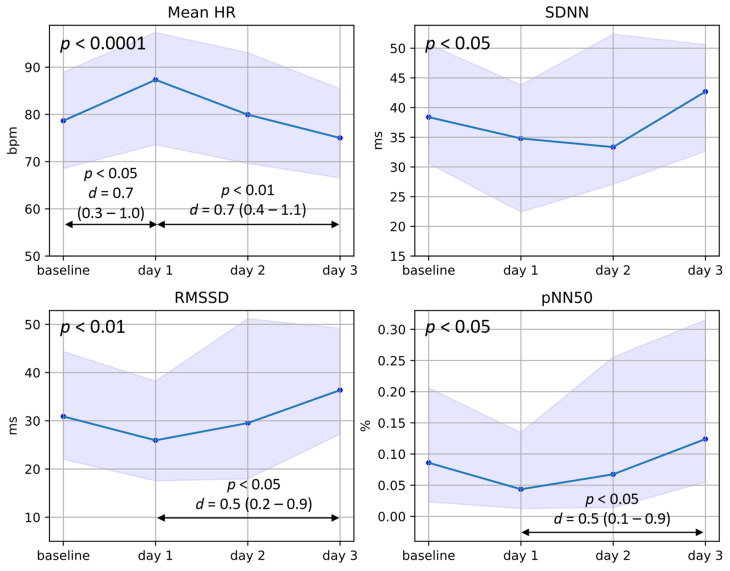
Plots of the median and interquartile ranges of maternal HR, SDNN, pNN50, and RMSSD in the days following betamethasone administration. The baseline represents measurements taken before the first injection of betamethasone or 96 h thereafter, while *day* 1 represents 24 h after the first injection, and *days* 2 and 3 represent 48 and 72 h thereafter, respectively. The statistical significance (*p*-value) for changes across the four days is presented in the top-left corner of each graph (from Friedman’s test), while differences between specific *days* are represented with arrows and corresponding *p*-values (from Dunn’s post hoc test) and *d*-values (Cohen’s effect size).

**Figure 5:**
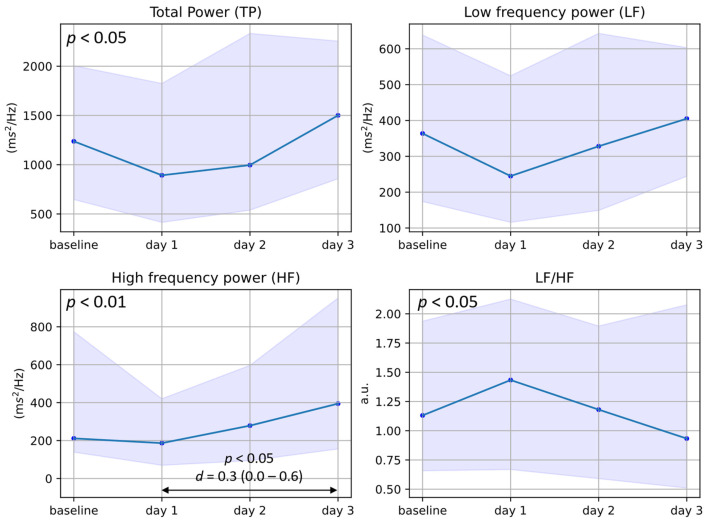
Plots of the median and interquartile ranges of maternal TP, LF, HF, and LF/HF in the days following betamethasone administration. The baseline represents measurements taken before the first injection of betamethasone or 96 h thereafter, while *day* 1 represents 24 h after the first injection, and *days* 2 and 3 represent 48 and 72 h thereafter, respectively. The statistical significance (*p*-value) for changes across the four days is presented in the top-left corner of each graph (from Friedman’s test), while differences between specific days are represented with arrows and corresponding *p*-values (from Dunn’s post hoc test) and *d*-values (Cohen’s effect size).

**Figure 6:**
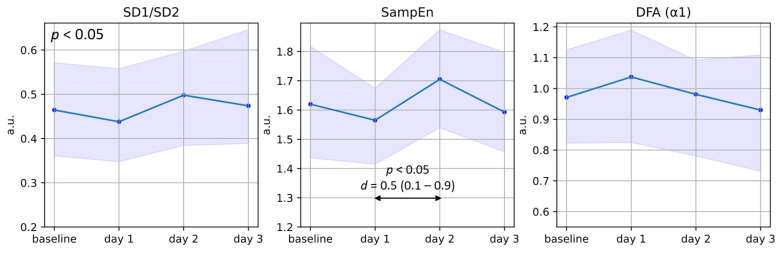
Plots of the median and interquartile ranges of maternal SD1/SD2, SampEn, and DFA α_1_ in the days following betamethasone administration. The baseline represents measurements taken before the first injection of betamethasone or 96 h thereafter, while *day* 1 represents 24 h after the first injection and *days* 2 and 3 represent 48 and 72 h thereafter, respectively. The statistical significance (*p*-value) for changes across the four days is presented in the top-left corner of each graph (from Friedman’s test), while differences between specific days are represented with arrows and corresponding *p*-values (from Dunn’s post hoc test) and *d*-values (Cohen’s effect size).

The results of the HR and the three sets of HRV features corresponding to the *baseline*, *day* 1, *day* 2, and *day* 3 are plotted in Figure 4, Figure 5 and Figure 6. The medians of the values are plotted, and the shaded area represents the interquartile ranges. The *p*-value of the overall significance of changes across the *days* is presented in the top-left corner of the graphs, while the statistical significance and effect size of differences between specific *days* are indicated with arrows at the bottom of the graphs. Of the included participants, 57 had data available for each *day* and were included in this section of the analysis. For the *baseline*, a median of 2.4% of IPIs were removed as specified in Section 2.2.3, while for *day* 1, *day* 2, and *day* 3, 3.6%, 3.2%, and 2.8% were removed, respectively.

#### 3.2.1. Mean HR and Time-Domain HRV Features

From Figure 4, we see that all time-domain features are significantly affected. HR increases significantly by about 10 bpm in the 24 h after the first betamethasone injections, with a mean *d* of 0.7 (note: *day* 1 is the time point preceding the second injection being administered). RMSSD and pNN50, which are features linked to parasympathetic activity, show medium to large decreases on *day* 1 compared to the baseline and a statistically significant decrease compared to *day* 3.

#### 3.2.2. Frequency-Domain HRV Features

In Figure 5, we see that TP, HF, and LF/HF change significantly change across the four *days*, while LF (which is linked to sympathetic activity) is not significantly altered. HF, which is parasympathetically modulated, was specifically significantly reduced on *day* 1 as compared to *day* 3.

#### 3.2.3. Non-Linear HRV Features

Of the non-linear or complexity features (Figure 6), only SD1/SD2 was found to change significantly across the four days, with a decrease on day 1 compared to baseline followed by an increase on day 2. Furthermore, SampEn shows a significant difference between day 1 and day 2. Note that a decrease in SD1/SD2 indicates similar physiological changes as an increase in LF/HF, namely increased long-term variation and/or decreased short-term variation.

### 3.3. Changes in Maternal Morphological Features Extracted from the PPG Waveform

The results from the unpaired analysis of the effect of betamethasone on morphological features are reported in this section. Note that an unpaired analysis was performed. The numbers of participants from which measurements (as defined in Section 2.3) were available for each *day* were as follows: 8 for *day* 0, 60 for *day* 1, 60 for *day* 2, 56 for *day* 3, and 53 for *day* 4.

**Figure 7:**
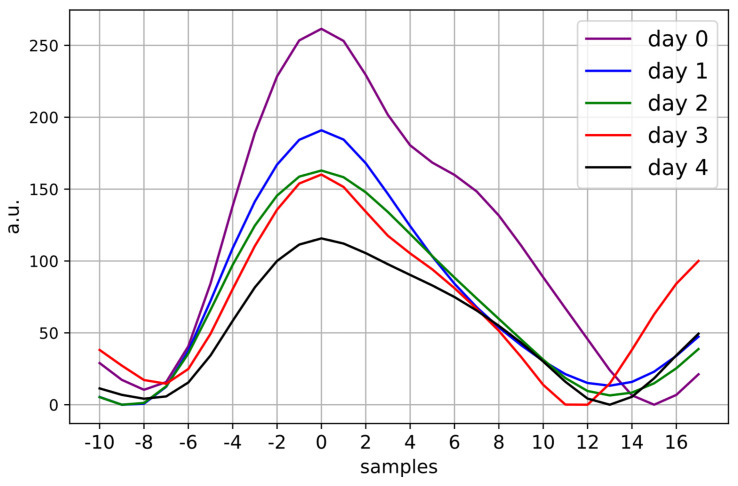
Ensemble averages of the PPG pulse waves analyzed for each day. The waveform becomes increasingly small from *day* 0 to *day* 4, with *day* 0 corresponding to the measurement before betamethasone is administered. *Day* 1, *day* 2, *day* 3, and *day* 4 correspond to measurements taken 24, 48, 72, and 96 h after the first betamethasone injection.

In Figure 7, the ensemble averages of all analyzed PPG pulse waves are plotted per *day*. Notice that the waveform becomes noticeably smaller from *day* 0 to *day* 4. However, the difference between day 0 and the other days may be overestimated as measurements from only eight participants are included for *day 0*, while over fifty are included for the other *days*. While no significant differences were found in the morphological features describing the area or amplitude of the PPG waveform, several other features had significant changes in the days following betamethasone administration. The angle between the IT and SP (α), SP/SPD, b2_amplitude, dw10/sw10, dw25/sw25, PSV, and FSV all decrease significantly across the five days, while t_a1 and t_a1/PWD increase significantly. The SPD also changes significantly, specifically with a statistically significant increase from *day* 1 to *day* 3 and *day* 4. The overall effect sizes of these changes were small to medium. Other features introduced in Table 2 showed no significant changes.

## 4. Discussion

Monitoring mHRV during pregnancy may offer novel opportunities for assessing maternal health and, in doing so, aid in the early detection of pregnancy complications. Facilitating such assessments requires not only an understanding of how pregnancy complications affect mHRV but also how other confounding factors may alter mHRV. A potential confounder that is often present when assessing mHRV in complicated pregnancies is the effect of antenatal corticosteroids. In this work, we show that corticosteroids—specifically, betamethasone—do seem to affect mHRV by increasing HR and decreasing HRV features linked to parasympathetic activity. Furthermore, we demonstrate for the first time, to our knowledge, how the PPG pulse wave morphology changes in response to corticosteroid administration.

Accurately assessing mHRV in women with pregnancy complications can be challenging. Women with complications such as threatened preterm birth, preterm rupture of membranes, or preeclampsia are typically admitted to the hospital upon diagnosis where they are administered medications such as corticosteroids with urgency. Therefore, acquiring measurements without the influence of such medications is not trivial. Some researchers avoid this by performing short measurements before corticosteroids are administered [9,40], while others mention these obstetric medications as potential confounders in their limitations [11,12]. Others still make no mention of corticosteroids, even though according to standard treatment protocols it is likely that their study groups would have been administered these medications [13,41,42,43]. Based on the results presented in this paper, we see that the effects of corticosteroids are not negligible, particularly in the 72 h following the first injection of betamethasone.

Consequently, it is important to consider how the administration of betamethasone may affect assessments of mHRV in women with pregnancy complications. Although the results on mHRV in this population presented in the literature are conflicting at times—potentially in part due to the influence of obstetric medications such as corticosteroids [10]—on average, pregnancy complications seem to be characterized by increased maternal sympathetic activity and decreased parasympathetic activity [44,45,46]. As corticosteroids seem to not affect sympathetic activity (see LF, Figure 5), likely, the increased sympathetic activity as captured by mHRV in complicated pregnancies is accurate. This increase is also confirmed by microneurography investigations, which directly measure sympathetic nerve activity [7,8]. However, care should be taken when interpreting studies showing reduced parasympathetic activity, as this could also result from or at least be amplified by the administration of corticosteroids. Considering the results of this study, along with the 36–59 h biological half-life of betamethasone [25,26], i.e., the half-life of its suppression of the hypothalamus–pituitary–adrenal (HPA) axis, we suggest that assessments of mHRV in complicated pregnancies should be performed either before or at least 72 h after the second betamethasone injection to minimize the confounding effect of this medication. However, it should be noted that we could not confirm how similar measurements are on *day* 0 and *day* 4, as only two participants had both measurements available.

In addition to the reduced parasympathetic activity, we also find that maternal HR is elevated by about 10 bpm (Figure 4) in the 24 h after the first betamethasone injection. Note that the measurement on *day* 1 is taken before the second injection of betamethasone is given; therefore, the increased HR is not a response to pain due to the injection. In recent work, we investigated the effect of corticosteroids on mHRV as determined from abdominal ECG measurements in a smaller group of hospitalized pregnant women [46]. In this previous work, we also found decreased parasympathetic activity along with a 10 bpm increase in HR in the 24 h after betamethasone administration—similar to the results observed in this work [46]. As vital signs such as maternal HR inform clinical decision-making [47,48,49], clinicians need to consider recent corticosteroid administration when evaluating maternal tachycardia.

In evaluating the results from the PPG pulse wave analysis (Figure 7), we find that several features reduce in the days following betamethasone administration. The effect of corticosteroids on the PPG pulse wave has not been previously demonstrated but the decreasing amplitude of the waveform seen in Figure 7 after betamethasone administration does appear to correspond to the vasoconstrictive effect of glucocorticoids—the class of steroids to which corticosteroids belong—on blood vessels [50]. Whether this has a wider impact on maternal physiology—for example, a corresponding increase in blood pressure—is currently unknown. But as in the case of HRV, the effects of corticosteroids on the PPG pulse wave seem to not be negligible.

The effects observed in mHRV and maternal PPG morphology likely result from the pharmacodynamics of betamethasone, specifically how this drug interacts with the human stress response. During the body’s stress response—typically partly mediated by the sympathetic nervous system—natural glucocorticoids are synthesized and these are perceived as cortisol which binds to glucocorticoid receptors, resulting in increased blood pressure (by way of vasoconstriction), increased HR, and decreased vagal tone [18]. Here, betamethasone (a synthetic glucocorticoid) administration leads to the same effect (Figure 4 and Figure 7). Note that as no natural glucocorticoids are synthesized, the sympathetic response appears not to be activated in this scenario (see LF in Figure 5). Once this increased level of (synthetic) glucocorticoids is detected in the bloodstream, the HPA axis is suppressed. The biological half-life of this suppression is 36–59 h [25,26]; subsequently, the effect of betamethasone should start to diminish on day 2 and day 3, as is confirmed by the decreasing HR and the increase in HRV features linked to parasympathetic activity (Figure 4 and Figure 5). Future work is needed to understand why HR seems to decrease and HRV seems to increase past the baseline on *day* 3. We speculate that on *day* 3 the effects of the HPA axis suppression may have worn off while the impact of the newly secreted cortisol is not yet seen in the HR and HRV features.

Furthermore, it is interesting that the opposite effect is observed in the mother than in the fetus. The literature reports that fetal HR typically decreases and HRV increases in the first 24 h after betamethasone administration [25,26]. However, it should be noted that the half-life of betamethasone in the mother is half of that in the fetus [51], meaning that the effects on the maternal and fetal systems are potentially occurring at different timescales. Still, future work is needed to understand whether this is indeed the case or whether the mother and fetus in fact have different physiological reactions to betamethasone administration.

This study has some limitations. Firstly, it is not possible to quantify the effect of the stress of the participants’ hospitalization on the results. Furthermore, the participants have different diagnoses and several of them were pregnant with multiples, which places an additional burden on their cardiovascular systems; to minimize the effects of these heterogeneities, a within-subject analysis was performed.

Moreover, several medications were co-administered to the participants as part of their standard treatment; this is unavoidable. Evaluating the impact of these medications on mHRV and PPG morphology in this study group is not feasible, as they are administered at different times and in different dosages. A dedicated future study would be necessary to determine the effect of these co-administered medications. However, the fact that we see a clear impact of corticosteroids on the outcome measures despite the heterogeneity of the participants increases our confidence that corticosteroids indeed affect maternal physiology. Furthermore, these results largely confirm those of previous work in our group, where we assessed the impact of corticosteroids on mHRV in a similar population, as calculated from abdominal ECG recordings [46]. In this previous work, we performed sub-analyses to explore the impact of additional obstetric medications. We did so by repeating our analysis, first excluding women who received nifedipine, which was the most commonly co-administered medication, and second excluding those using antihypertensives, which are known to affect mHRV [46]. In both cases, the trends in mHRV in the days following betamethasone administration were similar to those observed in the main analyses, which included all participants [46].

The reason for employing wrist-worn PPG data in this prospective study—as opposed to abdominal ECG as in our retrospective study mentioned above [46]—is that it was essential to use measurement technology which has minimal impact on clinical workflow in the obstetrics high-care unit [19]. Furthermore, these measurements further allow for studying the impact of corticosteroids on PPG morphology in addition to mHRV. However, here it should be noted that some researchers argue that the term pulse rate variability would be more appropriate given that HRV derived from PPG and the gold-standard measurement of ECG are not always comparable [52]. Still, others argue that they can be used as substitutes for each other given that the sampling rate is above 25 Hz [53], as is the case in this investigation. Consequently, we chose to use the term HRV in this work, which is also commonly used in the literature [5,54,55]. Still, no direct comparison of HRV and pulse rate variability has been performed in women with pregnancy complications to our knowledge.

Furthermore, using wrist-worn PPG data comes with some inherent limitations. PPG data are prone to motion artifacts and therefore, despite the large amount of data collected during this investigation, only five-minute measurements of sufficient quality were used for this analysis. Additionally, measurements were collected at different times of the day for different participants since corticosteroids are administered at varying times. To this end, we performed a within-subject analysis for the mHRV analysis to minimize the effect of circadian rhythms on the results. However, these differences will still affect the values of the HRV features presented in Figure 4, Figure 5 and Figure 6, as well as the results of the PPG pulse wave analysis. It should also be noted that posture changes can affect the amplitude of the PPG pulse wave, which may influence some of the features reported in Section 3.3. As this study setup mimics free-living conditions, we have no knowledge concerning the posture of the participants at the times of the measurements. However, these participants were on bed rest, meaning that likely they were in a semi-recumbent or supine position. Furthermore, any differences in posture are likely averaged out between participants during the analysis. The fact that significant changes across the days are still detected also provides additional evidence supporting our confidence that corticosteroids are affecting mHRV.

Still, future work is needed to confirm the results of this study, particularly in light of the heterogeneity of the study group. With the rising use of wearable abdominal ECG devices, it would be possible to repeat the mHRV aspect of this analysis with gold-standard ECG measurements, while maintaining a study design with minimal impact on clinical workflow. Additionally, a more extensive analysis of the PPG morphology may yield additional insights, particularly if PPG data are collected at a higher sampling rate. Given that this is a novel analysis of the impact of corticosteroids on PPG morphology, features commonly used in the literature (Table 2, Figure 3) and visual inspection (Figure 7) were employed. However, future studies should explore additional metrics such as those related to PPG waveform variability [56,57].

## 5. Conclusions

We believe this novel work is an important step towards a better understanding of how routine pharmaceuticals aimed at treating the fetus also affect maternal physiology. Our results indicate that administering corticosteroids increases maternal HR, decreases parasympathetic activity, and reduces indices that describe the morphology of the PPG pulse wave. The impact of corticosteroids on maternal physiology must be considered when investigating these features in the pregnant population.

## Figures and Tables

**Table 1:** Inclusion and exclusion criteria.

Inclusion Criteria	Exclusion Criteria
Age 18 years and above	History of severe arrhythmia and/or maternal congenital heart disease
Gestational age 23 5/7 to 33 6/7 weeks	Diseases with known effects on ANS
Yet to receive the second betamethasone injection	Known allergies to hard plastic (e.g., used in sport watches) or elastic band material
Proficient in Dutch or English	Wounds, injuries, or infectious diseases on the wrist where the PPG device will be worn
	Tattoo location on wrist interfering with the positioning of the PPG device
	Both wrists unavailable for wearing the PPG device (e.g., owing to intravenous lines)
	Dexamethasone (another brand of corticosteroid) administered instead of betamethasone

**Table 2:** Description of the morphological features. The fiducial points on the PPG pulse wave discussed in the table can be found in Figure 3. Note that in Figure 1, DW25 and SW25 can be found, which represent the diastolic and systolic widths at 25% of the amplitude. Where features such as DW are mentioned in the table, these are similar to DW25, but at 10% instead of 25%.

Features		Explanation
*Ampli-tude*	PWA	Pulse width amplitude, i.e., the difference between SP and IT.
	b2_amplitude	The absolute value of the amplitude of the deepest trough of the second derivative signal (b2)
*Time differences*	PWD	Pulse width duration; time interval between IT and FT
	SPD	Systolic phase duration; time interval between IT and SP
	DPD	Diastolic phase duration; time interval between SP and FT
	t_a1	Time interval between IT and a1 on the first derivative signal
	t_a1b1	Time interval between the a1 and the first valley of the first derivative signal (b1)
	t_a2b2	Time interval between points a2 and b2 on the second derivative signal
	t_b2e2	Time interval between points b2 and e2 on the second derivative signal
*AUC*	AUC_total	AUC of the full pulse wave, i.e., between IT and FT
	AUC1	AUC of systolic phase, i.e., between IT and SP
	AUC2	AUC of diastolic phase, i.e., between SP and FT
*Velocity and acceleration*	mean(V)	Mean velocity, i.e., mean of the first derivative signal
	IDR(V)	Interdecile range of velocity, i.e., interdecile range of the first derivative signal
	Mean (Acc)	Mean of the second derivative signal
	MSV	Max systolic velocity; a1 on the first derivative
	SFV	Systolic foot velocity, i.e., value of the point on the first derivative signal corresponding to IT of the pulse wave
*Ratio*	DW10/SW10	The ratio of systolic width to diastolic width at 10% of the pulse wave amplitude; similar features are calculated at 25%, 50%, and 60%.
	t_s/PWD	The ratio between the time interval between a1 and SP (i.e., t_s), and the pulse width duration (PWD), which is the time interval between IT and FT
	t_a1/PWD	The ratio of the time interval between the IT and a1 (i.e., t_a1) to PWD
	t_a1b1/PWD	The ratio of t_a1b1 to PWD
	t_a2b2/PWD	The ratio of t_a2b2 to PWD
	t_b2e2/PWD	The ratio of t_b2e2 to PWD
	b2/a2	The ratio of b2_amplitude to a2_amplitude, found on the second derivative signal
	e2/a2	The ratio of e2_amplitude to a2_amplitude, found on the second derivative signal
	SPD/PWD	The ratio of SPD to PWD
	SP/SPD	The ratio of the value of SP to SPD
	Pulsatility index	Max systolic velocity (i.e., a1 on the first derivative)–end diastolic velocity (i.e., EDV on the first derivative)/(mean of the first derivative)
*Slope*	slope_IT_SP	The slope of line that connects IT and SP
	slope_SP_FT	The slope of line that connects SP and FT
*Angle*	α	The angle of the slope between IT and SP
	γ	The angle of the slope between SP and FT

**Table 3:** Characteristics of the patients included in the study. Where applicable, values are presented as median with interquartile range.

Characteristic	
Indication for betamethasone (no. of participants)	
Threatened preterm labor	31
Vaginal bleeding	4
Preterm rupture of membranes	8
Preeclampsia and/or HELLP syndrome *	13
Pregnancy-induced hypertension	1
Fetal growth restriction	2
Non-obstetric operation	1
Suspicion of twin anemia polycythemia sequence	1
Gestational age at first betamethasone injection	28 weeks (26 weeks 3 days–30 weeks)
BMI (prepregnancy)	24.95 (22.05–27.70) kg/m^2^
Age	31 (27–33) years
Nulliparous	59.7%
Pregnancy with multiples	24.2%
Co-administration of medications during study period (no. of participants)	
Atosiban	11
Azitromycin	11
Nifidipine	19
Penicillen	2
Magnesium sulfate	19
Methyldopa	5
Labetalol	7

* HELLP = Hemolysis, Elevated Liver enzymes, and Low Platelets.

## Data Availability

The datasets used and/or analyzed during the current study are available from the corresponding author on reasonable request.

## Abbreviations

ANS	autonomic nervous system
HPA	hypothalamus–pituitary–adrenal
HR	heart rate
HRV	heart rate variability
IPI	inter-pulse interval
PPG	photoplethysmography
